# Effect of Yellow Wine Lees Supplementation on Milk Antioxidant Capacity and Hematological Parameters in Lactating Cows under Heat Stress

**DOI:** 10.3390/ani11092643

**Published:** 2021-09-09

**Authors:** Kaiyong Yao, Luyi Jiang, Jianxin Liu, Diming Wang, Hongyun Liu, Daxi Ren

**Affiliations:** 1 Institute of Dairy Science, College of Animal Sciences, Zhejiang University, Hangzhou 310058, China; 3100100217@zju.edu.cn (K.Y.); luyi08042@163.com (L.J.); liujx@zju.edu.cn (J.L.); wdm@zju.edu.cn (D.W.); hyliu@zju.edu.cn (H.L.); 2 Lanhai Ecological Agriculture (Hangzhou) Co., Ltd., Hangzhou 311402, China

**Keywords:** oxidative stress, fatty acids profile, milk stability, blood variables

## Abstract

**Simple Summary:**

The yellow wine lees (YWL), a byproduct of the yellow wine brewing industry which contain high levels of crude protein and anti-oxidative substrates can be a suitable ingredient in dairy rations. Total mixed rations (TMR) containing unfermented/fermented YWL were greater in total phenolic and flavonoid concentrations. Feeding lactating cows under heat stress with TMR containing unfermented or fermented YWL mix can reduce their inflammation response and oxidative stress, as well as improve fatty acid quality and oxidative stability of their milk.

**Abstract:**

Fifteen multiparous lactating Chinese Holstein dairy cows were used in a replicated 3 × 3 Latin Square Design to evaluate the effect of total mixed rations (TMR) containing unfermented and fermented yellow wine lees (YWL) on the oxidative status of heat-stressed lactating cows and the oxidative stability of the milk and milk fatty acids they produced. Cows were fed with three isonitrogenous and isocaloric diets as follows: (1) TMR containing 18% soybean meal, (2) TMR containing 11% unfermented YWL (UM), and (3) TMR containing 11% fermented YWL (FM). The rectal temperature (at 1300 h) and respiratory rate were higher in control cows than in cows fed UM or FM. Both types of YWL were greater in total phenolic and flavonoid contents, reducing power, and radical scavenging abilities than soybean meal. Cows fed UM or FM had higher blood neutrophil, white blood cell, and lymphocyte counts, as well as lower plasma malondialdehyde level, higher plasma superoxide dismutase, glutathione peroxidase, and 2,2-diphenyl-1-picryl-hydrazyl-hydrate levels, and higher total antioxidant capacity in the plasma than those fed control diet. The proportion of milk unsaturated fatty acids was higher and that of saturated fatty acids was lower in UM- and FM-fed animals than in the control animals. Milk from UM- and FM-fed cows had lower malondialdehyde content but higher 2,2-diphenyl-1-picryl-hydrazyl-hydrate content than the control cows. In conclusion, feeding TMR containing UM and FM to cows reduced both the oxidative stress in heat-stressed cows and improved the oxidative capacity of their milk.

## 1. Introduction

Heat stress leads to metabolic disorders in lactating dairy cows and reduces dry matter (DM) intake, milk production, and animal welfare [1,2,3]. In addition, it has been reported that cows exposed to heat stress showed decreased antioxidant enzyme activity which resulted in a higher level of free radicals and lower unsaturated fatty acid (UFA) and polyunsaturated fatty acid (PUFA) concentrations in their milk than non-stressed cows [4]. Thus, inflammatory diseases, such as mastitis and metritis, may be found in heat-stressed cows [5]. Additionally, milk containing lower levels of UFA (*cis*9, *trans*11-conjugated linoleic acid (CLA), 18:2n-6, and 18:3n-3) are prone to higher lipid peroxidation, which is associated with lower antioxidant capacities in milk [6]. Thus, it is necessary to consider how to maintain mammary gland health and milk stability in lactating cows under heat stress.

The antioxidant capacity of milk products is an important index for the dairy industry, as oxidation would induce deterioration of the milk nutritional quality [7]. Higher milk antioxidant content can efficiently prevent lipid peroxidation and extend the shelf life of milk [8]. Oxidation also leads to sensory quality deterioration, which can affect milk marketability [9]. Milk antioxidant capacity depends on various factors such as fatty acid compositions [6,10] and concentrations of other antioxidant substrates, such as phenolics and flavonoids [11]. Increasing dietary phenolic and flavonoid substrates induced by propolis extracts or *Agaricus blazei* mushrooms could enhance the milk antioxidant capacity of dairy cows, indicating that dietary intervention is useful in modifying milk antioxidant capacities [12,13]. Moreover, it has been reported that feeding lactating cows with dry distiller’s grains with solubles, which have high UFA proportions, improved milk UFA concentrations, but not milk antioxidant capacity [14,15,16]. This information indicated that the dairy rations, including microbiological products, may impact milk antioxidant capacity.

As a traditional Chinese alcoholic beverage, over one million tons of yellow wine lees (YWL) were produced from rice and wheat in 2015 [17,18]. We previously found that the antioxidant activity of the YWL can be improved when fermented with *Candida utilis* and *Bacillus subtilis* [19]. In our preliminary in vitro study, when the ratio of soybean meal (SBM) to unfermented (UM) or fermented YWL mix (FM) in the simulated ration was at 1:1 (DM basis), the gas production parameters and volatile fatty acids were optimized and functioned similarly to the rations with SBM as the main protein resource [20]. Moreover, we observed that total mixed rations (TMR) with partial replacement of SBM with UM/FM did not affect feed efficiency and milk compositions, indicating YWL as a potential protein source in dairy rations [21]. We hypothesized that milk antioxidant capacity is affected by the dietary application of UM/FM. To validate our hypothesis, this study was conducted to compare fatty acid profiles, antioxidant capacity-related indices in feed, blood, and milk, and hematological parameters in heat-stressed dairy cows fed with different daily rations.

## 2. Materials and Methods

### 2.1. Animal Care

Procedures concerning animal care and experimentation were approved by the Institutional Animal Care and Use Committee at Zhejiang University (Hangzhou, China, approval number 20041), which had met EU standards for the protection of animals and feed legislation.

### 2.2. In Vitro Antioxidant Capacity Assessment

The UM included YWL, wheat bran, cassava residue, and molasses at a ratio of 400, 500, 50, and 50 g/kg, respectively. A combination of two microorganisms, namely *C. utilis* KF01 (Accession No: 7691) and *B. subtilis* KF02 (Accession No: 7690), was applied to treat the UM through solid-state fermentation to produce FM [19]. The crude protein levels of UM and FM were 26.3% and 31.3% (DM basis), and the chemical compositions of UM and FM were reported in the previous study [19]. Chemical compositions of the three experimental TMR are listed in Supplementary Table S1, which has been published previously [21].

For the in vitro antioxidant capacity assessment, SBM, UM, and FM were sampled thrice with 70% (*v*/*v*) ethanol (1:10, *w*/*v*) at room temperature. The extracts were filtered, lyophilized, and kept at −20 °C for the investigation of total phenolic content, total flavonoid content, reducing power, superoxide radical scavenging ability, and hydroxyl radical scavenging ability. Total phenolic content was determined by the Folin–Ciocalteu assay using gallic acid (GA) as the standard [22]. Absorbance was measured at 765 nm using distilled water as blank. Results were expressed as GA equivalents (mg GA/g dried extract) [22]. Total flavonoid content was determined by a colorimetric method as described in the previous literature [22,23]. Results were expressed as catechin equivalents (mg catechin/g dried extract). Reducing power was determined according to the method of Oyaizu [24]. Superoxide radical scavenging activity was measured based on the method described by Robak and Gryglewski [25]. Hydroxyl radical scavenging activity was determined according to the method of Ardestani and Yazdanparast [22].

Evaluation of milk fatty acid compositions was conducted with the gas chromatography (GC) method (Model 2010, Shimadzu, Japan) as previously described [26]. A flame-ionization detector and a 100 m column with an i.d. of 0.25 mm (CP-Sil 88, Varian, Lake Forest, CA, USA) were equipped on the GC machine. A split ratio of 1:20 was applied on a split/splitless injector. Helium was used as the carrier gas, with the flow rate set to 2.0 mL/min. Samples were injected in GC conditions that were set as follows: initial temperature was 50 °C and was held for 1 min; then, the temperature was raised to 145 °C at a rate of 5 °C/min, and was held for 30 min. Afterwards, the temperature was then raised to 190 °C at a rate of 10 °C/min, and was held for 30 min. Lastly, temperature was raised to 210 °C at a rate of 5 °C/min, and was held for 35 min. Individual fatty acids are presented as g per 100 g of total fatty acids.

### 2.3. Animal Experimental Design

The animal experiment was conducted from July to September at the Zhengxing Dairy Farm (Hangzhou, China). Fifteen multiparous Chinese Holstein dairy cows (190 ± 15.2 days in milk) were employed in a replicated 3 × 3 Latin Square Design. Each period lasted 20 days. The first 15 days were treated as an adaptation period, and the following 5 days were for sampling. The three diets were as follows (DM basis): (1) 18% of SBM contained in the TMR (control), (2) 11% of unfermented YWL mix contained in the TMR (UM), and (3) 11% of FM contained in the TMR (FM). Details, including cost of the experimental rations, are described in Supplementary Table S1. The percentage for YWL feed inclusion was based on the former in vitro study [20] and the DMI and milk yield of FM was similar to the control diet, reported in the previous production study [21]. All of the diets were isonitrogenous and isocaloric, with a forage-to-concentrate ratio of 60:40 (DM basis), and met the requirement of net energy for lactation (NE_L_) producing 29 kg/day milk in dairy cows (Ministry of Agriculture of China, (Beijing, China), 2004) [21].

Cows were housed in a tie-stall barn and were fed and milked twice daily at 0600 and 1800 h. Feed was available *ad libitum* to allow for at least 5–10% orts, and all the cows had free access to drinking water within the experiment period.

### 2.4. Samplings and Analyses

To evaluate environmental conditions inside the barn, the temperature and relative humidity (RH) were recorded by calibrated data logging equipment (Ming Gao, Mingle Instruments Co. Ltd., Shenzhen, China) three times daily (07:00, 13:00, 19:00). Recorders were set at the middle of the study pen and were placed at a height of 1.8 m above the floor. The errors of the temperature and RH recorded were within ±0.2 °C and ±2%, respectively. The temperature–humidity index (THI) was calculated as follows: THI = dew point temperature (TD) − (0.55–0.55 RH/100) (TD − 58), wherein TD is the dry bulb temperature in °F (°F = 32 °C + 1.8 °C) and RH is expressed as a percentage [27]. Average daily temperature and RH were determined using the recording data, and mean THI values were then calculated. The error of the rectal temperatures recorded was within ±0.01 °C on the fifth day of each sampling period using clinical veterinary thermometers (Digital thermometers, Nulan Instruments Co. Ltd., Hebei, China) at 07:30 and 13:00, respectively. Respiratory rates were taken for 1 min with a stopwatch by counting flank movements [28]. Readings were taken for all cows at 07:30 and 13:00 in each sampling period.

Two milk samples were obtained on d 17 of each period. The first subsample of milk was used to analyze fatty acid profiles as previously described for feed fatty acid profile in the feed analysis section [26]. The second subsample was used to verify the concentrations of phenolics, flavonoids, and oxidative stress-related variables in milk. Concentrations of total phenolics and flavonoids were determined following the aforementioned methods [14,22]. Milk malondialdehyde (MDA) concentration was evaluated using the TBARS method (Nanjing Jiancheng Bioengineering Institute, Nanjing, China) [29]. The 2,2-Diphenyl-1-picrylhydrazyl (DPPH) scavenging activity was measured according to the method described by Li et al. [30].

Blood was collected from the coccygeal vein of each cow at about 3 h after feeding on d 17 of each period. One subsample was centrifuged at 3000× *g* for 15 min to obtain plasma, as previously reported [21]. The total protein, albumin, glucose, cholesterol, non-esterified fatty acid (NEFA), and β-hydroxybutyric acid (BHBA) of plasma samples were analyzed using an Auto Analyzer 7020 instrument (Hitachi High-technologies Corporation, Tokyo, Japan) with colorimetric commercial kits (Ningbo Medical System Biotechnology Co., Ltd., Ningbo, China). The MDA, superoxide dismutase (SOD), and glutathione peroxidase (GSH-Px), and the total antioxidant capacity (T-AOC) of plasma, were evaluated using commercial assay kits (Nanjing Jiancheng Bioengineering Institute, Nanjing, China) [31,32]. The DPPH scavenging activity was measured according to the method described by Li et al. [30]. Another set of subsamples collected into 2 mL ethylene diamine tetraacetic acid anticoagulant vacutainer tubes were used to immediately evaluate whole blood variables, including red blood cell, white blood cell (WBC), neutrophil, lymphocyte, monocyte, hematocrit, mean corpuscular volume, mean corpuscular hemoglobin, red cell distribution width, reticulocyte, eosnophils, basophil, platelets, mean platelet volume, platelet distribution width, and plateletcrit, using a hematology analyzer (IDEXX Sysmex Ltd., Chuo-ku, Kobe, Japan) at the affiliated animal hospital of Zhejiang University (Hangzhou, China).

### 2.5. Statistical Analysis

Statistical analysis was conducted using SAS software (version 9.2; SAS Institute, Inc., Cary, NC, USA). In vitro data were analyzed using the PROC ANOVA procedure. Data on animal experiment were analyzed using the PROC MIXED procedure in SAS software. The model included group, period, treatment, and interaction of treatment by group and interaction of treatment by period as fixed effects, and cow within the group was as a random effect. The results listed as least squares means were separated using the PDIFF option when the fixed effects were significant (*p* < 0.05), and *p* < 0.1 was defined as a statistical trend.

## 3. Results

**Figure 1:**
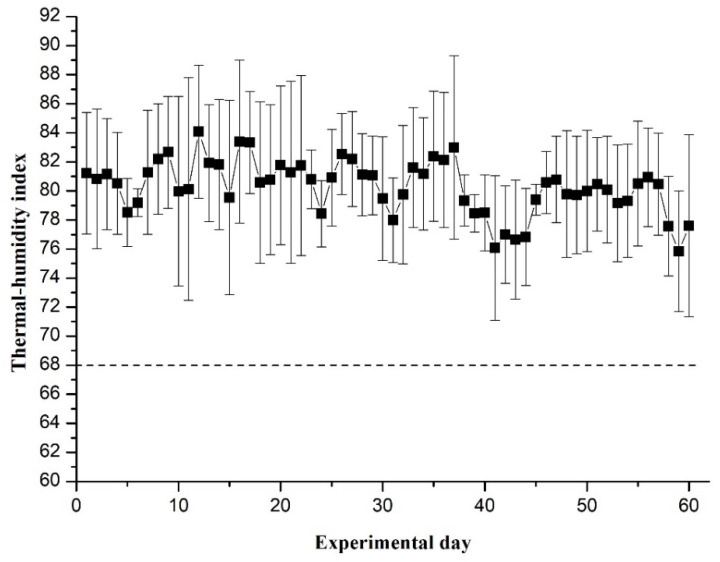
Thermal-humidity index of experimental days. The error bar stands for the standard error of thermal-humidity index of every experimental day.

Throughout the whole experiment, the average THI value was above 75 (Figure 1), indicating that all the experimental cows experienced heat stress throughout the experiment. Compared with control, the rectal temperature of cows fed UM and FM were similar in the morning (0700 h), however, they were lower in the afternoon (1300 h, *p* = 0.02, Table 1). Moreover, cows consuming UM and FM had lower respiratory rates relative to the control animals (*p* = 0.02, Table 1).

The phenolic and flavonoid contents, as well as the antioxidant activities of SBM, UM, and FM, are presented in Table 2. The UM and FM had greater total phenolics and total flavonoids relative to SBM (*p* < 0.01). Compared with UM, FM was higher in terms of total phenolic and flavonoid contents (*p* < 0.05). Reducing power, superoxide radical scavenging ability, and hydroxyl radical scavenging ability were greater in the UM and FM, compared to that of SBM (*p* < 0.01). Moreover, FM had similar reducing power of UM, but was greater in terms of superoxide radical scavenging ability and hydroxyl radical scavenging ability than UM (*p* < 0.01).

The plasma fatty acid compositions of cows in the three diet groups are presented in Table 3. Compared to control, UM and FM were higher in concentrations of C18:1 *cis*-9 (*p* = 0.02) and C18:1 *cis*-11 (*p* = 0.01), respectively. The concentrations of C22:4 and C22:5 n-3 in UM and FM were lower than in control (*p* = 0.01). Other fatty acid compositions did not differ across the three diets.

The milk fatty acid profiles of cows fed control, UM, and FM are presented in Table 4. Compared with control, cows fed UM or FM had greater proportions of C10:0, C18:0, C18:3 n-3, C20:0, and CLA (*cis*-9 *trans*-11) in their milk (*p* < 0.05). However, cows fed UM or FM had lower contents of C12:0, C14:0, C16:0, C16 *cis*-9, C17:0, C18:1 *trans*-6, C18:1 *cis*-9, and C19:0, compared to the control animals (*p* < 0.05). The short-chain fatty acids were not different among the treatments (*p* > 0.05), medium-chain fatty acids were lower, and long-chain fatty acids were higher in UM and FM animals than the control cows (*p* < 0.01). The SFA was lower in cows fed UM and FM, while UFA (mono- and poly-) was higher than the control cows (*p* < 0.01).

The plasma metabolites of experimental cows fed different diets are listed in Table 5. The concentrations of total protein, glucose, cholesterol, NEFA, and β-hydroxybutyric acid (BHBA) of plasma samples in the experimental cows were not affected by the treatments. The albumin concentration of cows tended (*p* = 0.07) to be affected by diet treatments. However, the numbers of the three groups were still close.

The whole blood variables of lactating cows fed different diets are listed in Table 6. In brief, cows fed FM and UM were lower in levels of WBC (*p* = 0.05) and neutrophil (*p* = 0.02) in their blood relative to the control animals. Similarly, blood lymphocyte concentrations tended to be lower in cows fed FM and UM relative to control (*p* = 0.07). Other hematological variables were similar among cows from the three treatment groups.

The antioxidant-related indices of the milk and plasma of cows from the three treatment groups are presented in Table 7. Cows fed with UM and FM had higher total phenolic and total flavonoid concentrations in milk than the control cows (*p* < 0.01). Higher levels of total phenolic and total flavonoid in milk and plasma of FM-fed animals were observed, compared with UM-fed ones (*p* < 0.05). Milk MDA concentration was lower in the UM- and FM-fed cows compared with control (*p* < 0.05). In contrast, milk DPPH concentrations were higher in cows with UM and FM compared to control (*p* < 0.01). In terms of the plasma, cows consuming UM and FM had higher total phenolic and total flavonoid concentrations relative to the control (*p* < 0.01). Concentrations of SOD (*p* = 0.03), GSH-PX (*p* = 0.04), and T-AOC (*p* = 0.01) were greater in UM- and FM-fed animals compared with control cows. In contrast, plasma MDA concentrations were lower in cows with UM and FM than in control cows (*p* = 0.05).

## 4. Discussion

Hydroxyl radicals and superoxide radicals are the most active forms of free oxygen in mammals [33]. Increasing the concentrations of antioxidant substrates, such as phenolics and flavonoids in the diet, could help improve antioxidant capacity of the animals and their milk [11]. Pretreatment with *Bacillus* has been reported to enhance total flavanol and phenolic acid concentrations in the soybean feed, which ultimately improves the DPPH radical scavenging ability of the fermented soybean feed [34]. Thus, a higher antioxidant capacity of UM and FM relative to SBM may be attributed to greater phenolic and flavonoid contents in TMR of the current study.

The daily THI throughout the experiment was above 68, which is the standard for exposure to heat stress for lactating dairy cows [27]. The lower respiratory rate and rectal temperature (measured at 1300 h) in cows that consumed UM and FM suggest that YWL supplementation may be beneficial to relieving heat stress in lactating dairy cows. Reduced heat stress induced by YWL could be attributed to the greater phenolic and flavonoid concentrations in the diets. Cows under heat stress produce a high level of harmful free radicals, such as reactive oxygen species, and exhibit a lower activity of antioxidant enzymes, which may induce an inflammatory reaction in dairy cows and enhance rectal temperature and respiratory rate [35]. Plant flavonoids, such as silymarin and quercetin, play important roles in improving antioxidant capacity in the CHO-K1 cell line [36]. It has been reported that flavonoids reduced endoplasmic reticulum stress and hepatic inflammation in early lactating dairy cows [37]. Moreover, polyphenols were reported to eliminate reactive oxygen species and improve the activity of antioxidant enzymes in dairy cows [38]. Dietary phenolics can reduce the inflammatory response in intestinal epithelial cells [39]. Thus, lower oxidative stress in cows fed UM and FM are partly attributed to increased polyphenol and flavonoid contents in the experimental feeds. Also, the higher inclusion of flavonoids and polyphenols in the FM diet caused a higher antioxidant component and capacity in the milk and plasma, compared with the UM diet. This is consistent with our previous production performance study, in which the DM intake, milk yield, and milk protein yield were higher in cows fed the FM diets than in cows fed the UM diet [21]. Min et al. [40] found that long-term heat stress can induce an inflammatory response in lactating cows, which can be induced by increased levels of WBCs, neutrophils, and lymphocytes in their blood. The relatively lower concentrations of WBC, neutrophil, and lymphocytes in the blood of cows fed with UM and FM suggested that the inflammation state of heat-stressed dairy cows had been relieved. In summary, partial replacement of SBM with UM or FM may benefit the health of dairy cows, especially in seasons or areas with a high THI.

Greater proportions of mono- and poly-UFA and lower proportions SFA in the milk of cows fed with UM and FM are related with greater UFA (such as C18:1 *cis*-9, C18:1 *Cis*-11, and C18:2) and numerically lower concentrations of some SFA (such as C16:0 and C20:0) in these animals, respectively. Secondly, phenolic components such as 4-methylcatechol can efficiently inhibit biohydrogenation in the rumen [41]. Feeding beef cows with TMR containing *Broussonetia papyrifera* L. silage improved PUFA in the meat, which is attributed to the inhibition of flavonoid-induced biohydrogenation in the rumen [42]. These results suggest that both phenolics and flavonoids can inhibit rumen biohydrogenation, which can potentially improve UFA concentrations in both blood and milk. Thus, relative to the control, the greater proportion of UFA in the milk fatty acids profile of cows consuming UM and FM is partly attributed to favorable fatty acid profiles and greater antioxidant molecules (phenolics and flavonoids) in the feed substrates.

A previous study suggested that the dietary addition of grape residue silage (containing high levels of phenolics and flavonoids) in the feed improved PUFA proportion, the PUFA to SFA ratio, and antioxidant capacity in the milk of dairy cows, which ultimately improved milk quality [43]. A recent finding also suggested that pomegranate peel addition, which contains high concentrations of polyphenols, can improve the milk antioxidant capacity of lactating ewes [44]. These observations suggested that food byproducts containing higher levels of phenolics or flavonoids could be beneficial to milk fat quality and shelf life. A higher SFA proportion in milk is associated with improved milk oxidative stability, while a higher milk UFA proportion may cause a lower oxidative stability of milk [4]. Aguiar et al. [12] found that milk with higher phenolic concentrations had greater oxidative stability even when it had a higher UFA proportion. In summary, our observations suggested that feeding cows with diets containing YWL could be beneficial to the oxidative stability of milk produced by cows during hot seasons, and it was found that the oxidative stability of milk in FM group was even higher.

## 5. Conclusions

During heat stress, partial replacement of soybean with UM/FM in the diets of mid-lactating dairy cows reduced oxidative stress, which is possibly associated with their increased levels of total phenolics and flavonoids. Moreover, UFA levels were increased when UM and FM were included in the diets. Due to the relatively high total phenolic and flavonoid concentrations in the YWL, milk from cows consuming these feeds were of high oxidative stability. The antioxidant component and capacity of plasma and milk were even higher in FM, indicating that the FM may have more potential to be utilized in dairy rations under heat stress. Further studies are still needed to investigate the oxidative off-flavors of milk from the different dairy rations, which may validate the findings of this study.

## Figures and Tables

**Table 1:** Rectal temperature and respiratory rate of lactating cows fed the total mixed rations (TMR) containing unfermented (UM) and fermented (FM) yellow wine lees (YWL) mix.

Items	Treatment ^1^			SEM	*p*-Value
	Control	UM	FM		
Rectal-Temperature					
07: 00 AM	38.6	38.7	38.5	0.08	0.39
13: 00 PM	39.0 ^a^	38.7 ^b^	38.6 ^b^	0.09	0.02
Respiratory rate	39.6 ^a^	36.2 ^b^	35.2 ^b^	1.68	0.05

^a,b^ Means within a row with different superscripts differ (*p* < 0.05); ^1^ Control = TMR containing soybean meal as main protein source; UM = TMR containing UM; FM = TMR containing FM; SEM = Standard Error of Mean.

**Table 2:** Total phenolic and flavonoid contents and in vitro antioxidant activity of soybean meal (SBM), and unfermented (UM) and fermented yellow wine lees mix (FM).

Items	SBM	UM	FM	SEM	*p*-Value
Total phenolic content, mg GAE/100 g	202 ^c^	582 ^b^	676 ^a^	16.3	<0.01
Total flavonoid content, mg CE/100 g	185 ^c^	273 ^b^	352 ^a^	6.25	<0.01
Reducing power	0.28 ^b^	0.76 ^a^	0.85 ^a^	0.04	<0.01
Superoxide radical scavenging ability, %	21.5 ^c^	64.1 ^b^	69.3 ^a^	1.78	<0.01
Hydroxyl radical scavenging ability, %	59.1 ^c^	70.5 ^b^	77.2 ^a^	1.25	<0.01

^a,b,c^ Means within a row with different superscripts differ (*p* < 0.05) SEM = Standard Error of Mean; GAE = garlic acid equivalents; CE = catechin equivalents.

**Table 3:** The plasma fatty acids compositions of cows fed the total mixed rations (TMR) containing unfermented (UM) and fermented yellow wine lees mix (FM).

Compositio %	Treatment ^1^			SEM	*p*-Value
	Control	UM	FM		
C12:0	3.73	3.81	3.78	0.069	0.74
C14:0	4.15	4.12	4.16	0.057	0.82
C14:1	0.44	0.26	0.40	0.556	0.13
C16:0	20.3	19.0	18.3	0.681	0.18
C16:1	0.30	0.15	0.23	0.073	0.11
C18:0	2.71	2.35	2.49	0.130	0.23
C18:1 *cis*-9	8.5 ^b^	10.7 ^a^	10.7 ^a^	0.453	0.02
C18:1 *cis*-11	36.7 ^b^	39.9 ^a^	40.1 ^a^	0.60	0.01
C18:2	5.91	6.40	6.27	0.137	0.10
C18:3n-3	0.53	0.48	0.47	0.016	0.07
C18:3n-6	3.54	2.76	2.83	0.237	0.11
C20:0	3.54	2.76	2.84	0.237	0.11
C20:1	0.67	0.38	0.65	0.092	0.09
C20:2	0.18	0.11	0.17	0.027	0.22
C20:5	0.50	0.50	0.49	0.046	0.98
C22:0	0.40	0.22	0.34	0.047	0.08
C22:5 n-3	9.97 ^a^	7.59 ^b^	7.30 ^b^	0.388	<0.01
C22:4	0.23 ^a^	0.14 ^b^	0.18 ^b^	0.015	0.01
C22:6	0.51	0.53	0.52	0.070	0.98
C24:0	0.10	0.05	0.08	0.011	0.08
C24:1	0.22	0.14	0.15	0.058	0.64

^a,b^ Means within a row with different superscripts differ (*p* < 0.05) ^1^ control = TMR containing soybean meal as main protein source; UM = TMR containing UM; FM = TMR containing FM; SEM = Standard Error of Mean.

**Table 4:** Milk fatty acids compositions of lactating cows fed the total mixed rations (TMR) containing unfermented (UM) and fermented yellow wine lees mix (FM).

Composition, %	Treatment ^1^			SEM	*p*-Value
	CON	UM	FM		
C4:0	3.17	2.83	2.96	0.127	0.17
C6:0	1.79	1.96	1.91	0.092	0.24
C8:0	1.42	1.28	1.22	0.061	0.09
C10:0	2.60 ^b^	2.95 ^a^	3.09 ^a^	0.088	<0.01
C12:0	4.31 ^a^	3.82 ^b^	3.47 ^b^	0.191	<0.01
C12:1	0.36	0.35	0.24	0.072	0.16
C14:0	14.6 ^a^	10.4 ^b^	10.3 ^b^	0.528	<0.01
C14:1	0.45	0.44	0.30	0.082	0.13
C15:0	0.62	0.61	0.48	0.086	0.20
C16:0	26.9 ^a^	21.1 ^b^	22.0 ^b^	0.860	<0.01
C16:1 *cis*-9	2.97 ^a^	1.65 ^b^	1.73 ^b^	0.171	<0.01
C17:0	0.49 ^a^	0.40 ^a,b^	0.31 ^b^	0.049	0.04
C17:1 *cis*-9	0.27	0.30	0.20	0.047	0.15
C18:0	7.64 ^b^	9.89 ^a^	10.1 ^a^	0.23	<0.01
C18:1 *trans*-6	0.60 ^a^	0.59 ^a^	0.40 ^b^	0.064	0.03
C18:1 *trans*-10	0.548	0.564	0.547	0.0639	0.24
C18:1 *trans*-11	1.22	1.32	1.26	0.120	0.20
C18:1 *cis*-9	30.8 ^a^	28.8 ^b^	29.9 ^a,b^	0.64	<0.01
C18:1 *cis*-11	0.58	0.59	0.62	0.049	0.18
C18:2	1.19	1.39	1.23	0.085	0.20
C18:3 n-3	0.28 ^b^	0.40 ^a^	0.41 ^a^	0.013	<0.01
C18:3 n-6	0.091	0.098	0.093	0.0131	0.93
C19:0	0.37 ^a^	0.30 ^a,b^	0.23 ^b^	0.039	0.02
C20:0	0.095 ^b^	0.134 ^a^	0.151 ^a^	0.0069	<0.01
C20:1 *trans*-11	0.31	0.36	0.21	0.072	0.08
CLA (*cis*-9 *trans*-11)	0.52 ^b^	1.17 ^a^	1.39 ^a^	0.068	<0.01
C20:0	0.52	0.48	0.39	0.092	0.26
C24:0	0.54	0.49	0.42	0.081	0.24
Short	6.38	6.07	6.03	0.149	0.22
Medium	51.9 ^a^	40.4 ^b^	41.5 ^b^	0.982	<0.01
Long	41.7 ^b^	53.6 ^a^	52.5 ^a^	0.853	<0.01
SFA	65.0 ^a^	56.8 ^b^	57.4 ^b^	0.827	<0.01
MUFA	31.1 ^b^	37.7 ^a^	36.9 ^a^	0.892	<0.01
PUFA	3.84 ^b^	5.48 ^a^	5.63 ^a^	0.103	<0.01

^a,b^ Means within a row with different superscripts differ (*p* < 0.05) ^1^ control = TMR containing soybean meal as main protein source; UM = TMR containing UM; FM = TMR containing FM; SEM = Standard Error of Mean.

**Table 5:** Plasma metabolites in dairy cows fed the experimental total mixed rations (TMR) containing unfermented (UM) and fermented yellow wine lees mix (FM).

Item	Treatment ^1^			SEM	*p*-Value
	Control	UM	FM		
Total protein, g/L	73.3	72.8	72.3	1.13	0.51
Albumin, g/L	30.0	31.3	30.8	0.43	0.07
Glucose, mmol/L	3.31	3.33	3.39	0.10	0.84
Cholesterol, mmol/L	5.10	5.16	4.85	0.21	0.20
NEFA ^2^, μM/L	197	176	168	14.6	0.15
BHBA ^3^, μM/L	540	590	584	21.1	0.22

^1^ Control = TMR containing soybean meal as the main protein source; UM = TMR containing unfermented YWL mix; FM = TMR containing fermented YWL mix; SEM = Standard Error of Mean ^2^ NEFA = non-esterified fatty acid ^3^ BHBA = β-hydroxybutyric acid.

**Table 6:** Whole blood variables of dairy cows fed the total mixed rations (TMR) containing unfermented (UM) and fermented yellow wine lees mix (FM).

Items ^1^	Treatments ^2^			SEM	*p*-Value
	Control	UM	FM		
Red blood cell, 10^6^/μL	6.37	6.60	6.37	0.13	0.10
White blood cell, 10^3^/μL	15.1 ^a^	13.4 ^b^	13.0 ^b^	1.02	0.05
Neutrophil, 10^3^/μL	4.59 ^a^	3.86 ^b^	3.63 ^b^	0.32	0.02
Lymphocyte, 10^3^/μL	8.70	7.99	7.46	0.49	0.07
Monocyte, 10^3^/μL	1.33	1.20	1.27	0.16	0.80
Hematocrit, %	28.4	29.7	28.7	0.70	0.13
Mean corpuscular volume, fL	44.6	45.1	45.1	0.56	0.28
MCH ^1^, pg	15.9	16.0	16.0	0.18	0.48
Red cell distribution width,%	27.2	27.4	28.3	0.56	0.11
Reticulocyte, 10^3^/μL	1.18	1.15	1.24	0.24	0.90
Eosnophils, 10^3^/μL	0.43	0.46	0.48	0.10	0.42
Basophil, 10^3^/μL	0.008	0.007	0.007	0.002	0.94
Platelets, 10^3^/μL	330	325	357	15.2	0.32
Mean platelet volume, fL	6.29	6.35	6.37	0.12	0.34
Platelet distribution width, fL	7.80	7.67	7.76	0.26	0.59
Platelet crit, %	0.21	0.20	0.23	0.02	0.24

^a,b^ Means within a row with different superscripts differ (*p* < 0.05) ^1^ MCH = average hemoglobin content of red blood cells; fL = μm^3^, ^2^ control = TMR containing soybean meal as main protein source; UM = TMR containing UM; FM = TMR containing FM; SEM = Standard Error of Mean.

**Table 7:** Redox variables in plasma and milk of lactating cows fed the total mixed rations (TMR) containing unfermented (UM) and fermented yellow wine lees mix (FM).

Items ^1^	Treatment ^2^			SEM	*p*-Value
	Control	UM	FM		
Milk					
Phenolic, mg GAE/mL	0.276 ^c^	0.429 ^b^	0.654 ^a^	0.229	<0.01
Flavonoid, mg CE/mL	0.188 ^c^	0.281 ^b^	0.426 ^a^	0.214	<0.01
MDA, uM	99.5 ^a^	70.2 ^b^	61.9 ^b^	3.41	<0.01
DPPH, U/mL	72.2 ^b^	100.6 ^a^	104.9 ^a^	3.71	<0.01
Plasma					
Phenolic, mg GAE/mL	0.064 ^c^	0.078 ^b^	0.144 ^a^	0.0061	<0.01
Flavonoid, mg CE/mL	0.043 ^c^	0.064 ^b^	0.093 ^a^	0.0047	<0.01
DPPH, U/mL	146.6 ^b^	190.9 ^a^	175.2 ^a^	9.91	0.02
SOD, U/mL	81.7 ^b^	86.9 ^a^	87.7 ^a^	2.16	0.03
GSH-Px, U/mL	162 ^b^	169 ^a,b^	175 ^a^	3.45	0.04
MDA, nmol/mL	5.30 ^a^	4.87 ^a,b^	4.32 ^b^	0.38	0.05
T-AOC, U/mL	1.78 ^c^	2.01 ^b^	2.25 ^a^	0.12	0.01

^a,b,c^ Means within a row with different superscripts differ (*p* < 0.05) ^1^ GAE = garlic acid equivalents; CE = catechin equivalents; MDA = malondialdehyde; DPPH = 2,2-diphenyl-1-picryl-hydrazyl-hydrate; SOD = superoxide dismutase; GSH-Px = glutathione peroxidase; T-AOC = total antioxidant capacity. ^2^ Control = TMR containing soybean as main protein source; UM = TMR containing UM; FM = TMR containing FM; SEM = Standard Error of Mean.

## Data Availability

All data are included in the article.

## Supplementary Materials

The following are available online at https://www.mdpi.com/article/10.3390/ani11092643/s1, Table S1: Components and chemical composition of the experimental diets.
