# Supplementary material for: Effect of Yellow Wine Lees Supplementation on Milk Antioxidant Capacity and Hematological Parameters in Lactating Cows under Heat Stress
Source: Animals (Basel). 2021 Sep 9;11(9):2643. doi: 10.3390/ani11092643 (PMC8468981; doi:10.3390/ani11092643)
Supplement: Supplementary file 1 [file animals-11-02643-s001.zip › animals-1348314-supplementary.pdf]

Supplementary Table S1. Components and chemical composition of the experimental diets

Items	Treatment[1]		
	Control	UM	FM
Dry matter (DM), %	46.1	46.0	46.1
Ingredients, % DM			
Alfalfa hay	14.45	20.00	20.49
Oat hay	23.64	16.89	14.19
Corn silage	23.8	22.98	25.3
Ground corn	4.44	8.36	9.11
Soybean meal	17.74	10.86	9.20
Rapeseed meal	0.37	0.90	0.98
Yellow wine lees mix	0	10.56	0
Fermented YWL	0	0	11.05
Beet pulp	13.6	7.47	7.74
Fat powder	0.30	0.51	0.56
Premix[2]	1.69	1.47	1.38
Composition, % DM			
Crude protein	16.16	16.12	16.20
Neutral-detergent fiber	34.5	35.6	34.7
Acid-detergent fiber	18.3	18.9	19.2
Ether extract	3.6	3.8	3.8
Nonfiber carbohydrate [3]	38.2	37.0	37.3
Ca	0.68	0.72	0.70
P	0.48	0.49	0.46
RDP[4], % of CP	52.4	50.3	52.5
RUP[5], % of CP	47.6	49.7	47.5
NE_L[6], Mcal/kg	1.66	1.67	1.67
Diet Price[7], $/kg of DM	0.387	0.364	0.363

[1]Control = total mixed ration containing SBM as main protein source; UM = total mixed ration containing YWL mix; FM = total mixed ration containing fermented YWL mix.

[2]Contained (per kilogram of DM): 250,000 IU of vitamin A; 50,000 IU of vitamin D; 1,400 IU of vitamin E; 600 mg of Fe; 650 mg of Cu; 3,000 mg of Zn; 630 mg of Mn; 17 mg of Se; 36 mg of I; 8 mg of Co; and 150-180 g of NaCl.

[3]Nonfiber carbohydrate = 100 − % Neutral-detergent fiber − % Crude protein − % Ether extract − % Ash.

[4]Rumen degradable protein, determined following the nylon bag method (Ørskov et al., 1980).

[5]Rumen undegradable protein (%) = 100 − % RDP.

[6] Calculated based on China recommendations (MOA, 2004).

[7] Diet price was calculated based on the ingredient price when purchased by the farm.
